# Author Correction: Strong indirect coupling between graphene-based mechanical resonators via a phonon cavity

**DOI:** 10.1038/s41467-019-09240-8

**Published:** 2019-03-19

**Authors:** Gang Luo, Zhuo-Zhi Zhang, Guang-Wei Deng, Hai-Ou Li, Gang Cao, Ming Xiao, Guang-Can Guo, Lin Tian, Guo-Ping Guo

**Affiliations:** 1 CAS Key Laboratory of Quantum Information, University of Science and Technology of China, Hefei, 230026 Anhui China; 2 Synergetic Innovation Center of Quantum Information and Quantum Physics, University of Science and Technology of China, Hefei, 230026 Anhui China; 3 School of Nature Sciences, University of California, Merced, CA 95343 USA

Correction to: *Nature Communications* 10.1038/s41467-018-02854-4; published online 26 January 2018

The original version of this Article contained an error in the last sentence of the second paragraph of the ‘Raman-like coupling between well-separated resonators’ section of the Results, which incorrectly read ‘On the contrary, when the detuning Δ_12_ is lowered to ~180 kHz, a distinct avoided level crossing between modes R_1_ and R_3_ is observed, as shown inside the dashed circle in Fig. 2b.’ The correct version states ‘Δ_12_/2π’ in place of ‘Δ_12_.’

Additionally, the first sentence of the ‘Theory of three-mode coupling’ section of the Methods originally incorrectly read ‘We describe this three-mode system with the Hamiltonian (*ħ* = 4).’ The correct version states ‘*ħ* = 1’ instead of ‘*ħ* = 4’.

Equation 8 was originally missing a factor of ‘*a*_2_’ from the second term, and incorrectly read:

$$a_{{\mathrm{\Delta }} \pm } = \frac{{({\mathrm{\Omega }}_{12}a_1 \pm \left( {\omega _{\Delta 0} \mp \Delta } \right) + {\mathrm{\Omega }}_{23}a_3)}}{{\sqrt {2\omega _{{\mathrm{\Delta }}0}(\omega _{{\mathrm{\Delta }}0} \mp \Delta )} }}.$$

The correct form of Equation 8 is:

$$a_{{\mathrm{\Delta }} \pm } = \frac{{({\mathrm{\Omega }}_{12}a_1 \pm \left( {\omega _{\Delta 0} \mp \Delta } \right)a_2 + {\mathrm{\Omega }}_{23}a_3)}}{{\sqrt {2\omega _{{\mathrm{\Delta }}0}(\omega _{{\mathrm{\Delta }}0} \mp \Delta )} }}.$$

The eleventh and twelfth sentences of the second paragraph of the ‘Theory of three-mode coupling’ section of the Methods originally incorrectly read ‘The nearly degenerate modes can be viewed as a hybridization of *α*_1_ and *α*_3_ with an effective coupling ${\mathrm{\Omega }}_{13}\left( {{\mathrm{\alpha }}_1^\dagger {\mathrm{\alpha }}_3 + {\mathrm{\alpha }}_3^\dagger \alpha _1} \right){\mathrm{/}}2$. The magnitude of this effective coupling is equal to the frequency splitting between the nearly degenerate modes, with ${\mathrm{\Omega }}_3 = ({\mathrm{\Omega }}_{12}^2 + {\mathrm{\Omega }}_{23}^2){\mathrm{/}}2{\mathrm{\Delta }}$.’ These sentences have been replaced with ‘The nearly degenerate modes can be viewed as a hybridization of *α*_1_ and *α*_3_ with an effective splitting $({\mathrm{\Omega }}_{12}^2 + {\mathrm{\Omega }}_{23}^2){\mathrm{/}}2{\mathrm{\Delta }}$.’

In Equation 10, the term on the left-hand side was originally incorrectly given as ‘*M*’. The corrected version changes this to ‘*M*_eff_’.

Finally, the original version of this Article contained an error in Fig. 3b, in which the units on the *y*-axis were incorrectly given as ‘MHz’, rather than the correct ‘kHz’.

This has been corrected in both the PDF and HTML versions of the Article.

