# Supplementary material for: Strong indirect coupling between graphene-based mechanical resonators via a phonon cavity
Source: Nat Commun. 2018 Jan 26;9:383. doi: 10.1038/s41467-018-02854-4 (PMC5786116; doi:10.1038/s41467-018-02854-4)
Supplement: Supplementary file 1 — Supplementary Information [file 41467_2018_2854_MOESM1_ESM.pdf]

Supplementary Methods

A. Sample fabrication

Supplementary Figure 1(a) shows the SEM photograph of a typical sample, with three suspended graphene resonators in an array. The cross-section schematic is shown in Supplementary Figure 1(b). The detailed sample fabrication process is as follows. After a step of E-beam Lithography (EBL), three parallel trenches are etched into a SiO_2 layer, which covers a highly resistive silicon wafer by reactive ion etching with a depth of 150 nm. The widths of the trenches are designed to be 2 μm. After a second step of EBL with precise alignment, 5 nm titanium and 30 nm gold are evaporated onto the wafer to form both the contacts and the bottom gates simultaneously. Both end contacts (contacts 1 and 4) are designed to be 2 μm, while the contacts 2 and 3 are 1 μm in widths. The widths of the bottom gates are designed to be 1.2 μm. Finally, the graphene ribbon, exfoliated on a polydimethylsiloxane (PDMS) stamp, is aligned and transferred over the trenches [1].

B. Measurement setup

As shown in Supplementary Figure 2, we name the four contacts as Source (S), Drain 1 ($D1$), Drain 2 ($D2$) and Drain 3 ($D3$). Typically, the probe microwave field can be applied to the source and detected at the drain electrodes. The suspended graphene is biased and actuated by the three bottom electrodes ($g1$, $g2$ and $g3$) underneath the ribbon (Supplementary Figure 2). The gate voltages, V_{gi}, induce

an additional charge $\langle q_i \rangle = C_{gi} V_{gi}$ $(i = 1, 2, 3)$ on the graphene, where C_{gi} is the gate capacitance of the i-th graphene resonator. The attraction between the charge q_i and its opposite charge $-q_i$ on the i-th gate causes an electrostatic force downward on the graphene resonator, leading to a mean electrostatic force

$$F_i = \frac{\partial \langle U_i \rangle}{\partial z_i} = \frac{1}{2} \frac{\partial C_{gi}}{\partial z} \left(V_{gi}^{\mathrm{DC}} + \delta V_{gi}\right)^2. \qquad (1)$$

Here, $\frac{\partial C_{gi}}{\partial z}$ is the derivative of the gate capacitance with respect to the distance between the gates and the graphene, while V_{gi}^{DC} and δV_{gi} are the DC and AC voltages applied to the electrodes, respectively.

By applying a DC voltage, V_{gi}^{DC}, the beam-like electromechanical resonator can be deformed by the static force $F_i^{\mathrm{DC}} = \frac{1}{2} \frac{\partial C_{gi}}{\partial z} \left(V_{gi}^{\mathrm{DC}}\right)^2$, , and the induced tension on the graphene changes the frequencies of the mechanical resonator.

If an RF voltage $\delta V_{gi}^{\mathrm{RF}} \cos\left(2\pi f_{gi} t\right)$ is applied with the frequency $f_{gi} = \omega_i / 2\pi$ approaching the resonant frequency $f_{mi} = \omega_{mi} / 2\pi$ of the i-th resonator, the periodic driving force $F_i^{\mathrm{AC}} = \frac{\partial C_{gi}}{\partial z} V_{gi}^{\mathrm{DC}} \delta V_{gi}(t)$ will actuate the mechanical vibration. Phonons can also be generated by a microwave driving force $F_i^{\mathrm{para}} = \frac{1}{2} \frac{\partial C_{gi}}{\partial z} \left(\delta V_{gi}^{\mathrm{RF}}\right)^2 \cos^2\left(2\pi f_{gi} t\right)$ with $f_{gi} = f_{0i}/2$.

To detect the resonance properties, another microwave tone with a frequency $\omega + \delta\omega$ was applied to contact *S* and was detected at contact *D*3 after mixing with another driving tone with frequency ω from the bottom gates. In this case, $\delta V_{sd}(t) = \delta V_{sd}^{\mathrm{RF}} \cos[(\omega + \delta\omega)t]$ is transferred across all three resonators, and is mixed with the driving fields from each bottom gate. The final mixing current is given by the product of $\delta V_{sd}(t)$ and the total modulated conductance δG:

$$I_{mix}(\omega, t) = \delta V_{sd}(t) \delta G, \qquad (2)$$

where the modulated conductance is defined by $1/(\delta G) = \sum 1/(\delta G_i)$, and δG_i can be defined as follows. Near the chosen gate voltage:

$$G_i \approx G_i\left(V_{gi}^{\mathrm{DC}}\right) + \frac{dG_i}{dz_i}\delta z_i = G_i\left(V_{gi}^{\mathrm{DC}}\right) + \frac{dG_i}{dn_i}\frac{dn_i}{dz_i}\delta z_i. \qquad (3)$$

With $n_i = C_{gi}V_{gi}/A_i$, and A_i being the area of the i-th suspended graphene section. Then we have:

$$\frac{dn_i}{dz_i} = \frac{1}{A}\frac{d(C_{gi}V_{gi})}{dz_i} \approx \frac{1}{A}V_{gi}\frac{dC_{gi}}{dz_i}, \qquad (4)$$

and

$$\delta G_i = \frac{V_{gi}}{C_{gi}}\frac{dG_i}{dV_{gi}}\frac{dC_{gi}}{dz_i}\delta z_i. \qquad (5)$$

Supplementary Note 1: Tunability of the mechanical resonators

Supplementary Figure 3 shows the broadband tunability of the resonant frequency for resonators R_1 and R_2, while the results of resonator R_3 has been shown in the main text. The frequencies linearly increase with the corresponding gate voltage (for positive gate voltage). This can be explained as an incremental increase of the elastic tension on the graphene, which is almost linearly proportional to the perturbative DC voltage applied to the gate. We extract the coefficient $df_{01}/d\,V_{g1}^{\mathrm{DC}} \sim 7\ \mathrm{MHz/V}$, $df_{02}/d\,V_{g2}^{\mathrm{DC}} \sim 6.7\ \mathrm{MHz/V}$, and $df_{03}/d\,V_{g3}^{\mathrm{DC}} \sim 7.7\ \mathrm{MHz/V}$ for the fundamental modes of each resonator. These numbers are in consistent with the results in previous reports on graphene systems[2].

Supplementary Note 2: Two-mode model

To quantitatively verify the phonon-phonon interaction mechanism of neighboring resonators in Fig. 1(d,e) of the main text, we model the system using the Hamiltonian ($\hbar = 1$)

$$\mathcal{H}_m = \omega_{m1}\alpha_1^*\alpha_1 + \omega_{m2}\alpha_2^*\alpha_2 + \frac{\Omega_{12}}{2}(\alpha_1^*\alpha_2 + \alpha_1\alpha_2^*), \qquad (6)$$

where Ω_{12} is the phonon hopping rate between these resonators. Because of this coupling, the normal modes are a hybridization of the two resonator modes. To be noted, the description is in analogy with the common practice used in quantum optics, but the system described here is in classical regime. The frequencies of the normal modes are

$$\omega_{\pm} = \frac{1}{2}(\omega_{m1} + \omega_{m2} \pm \omega_0), \qquad (7)$$

where $\omega_0 = \sqrt{\delta^2 + \Omega^2}$ corresponds to the frequency splitting between the normal modes and $\delta = \omega_{m1} - \omega_{m2}$ is the frequency difference between the mechanical modes. The normal modes can be written as

$$A_{\pm} = \sqrt{\frac{\omega_0 \pm \delta}{2\omega_0}}\alpha_1 \pm \sqrt{\frac{\omega_0 \mp \delta}{2\omega_0}}\alpha_2, \qquad (8)$$

which are superpositions of the original mechanical modes. At $\delta = 0$, $A_{\pm} = (\alpha_1 \pm \alpha_2)/\sqrt{2}$, and are equal superpositions of the mechanical modes; and the mode splitting is at its narrowest with $\omega_+ - \omega_- = \Omega$. By varying the frequency difference of the two mechanical modes, the normal modes can be tuned. Supplementary Figure 4(a) shows the results calculated from the theory, and (b) shows the corresponding experiment results.

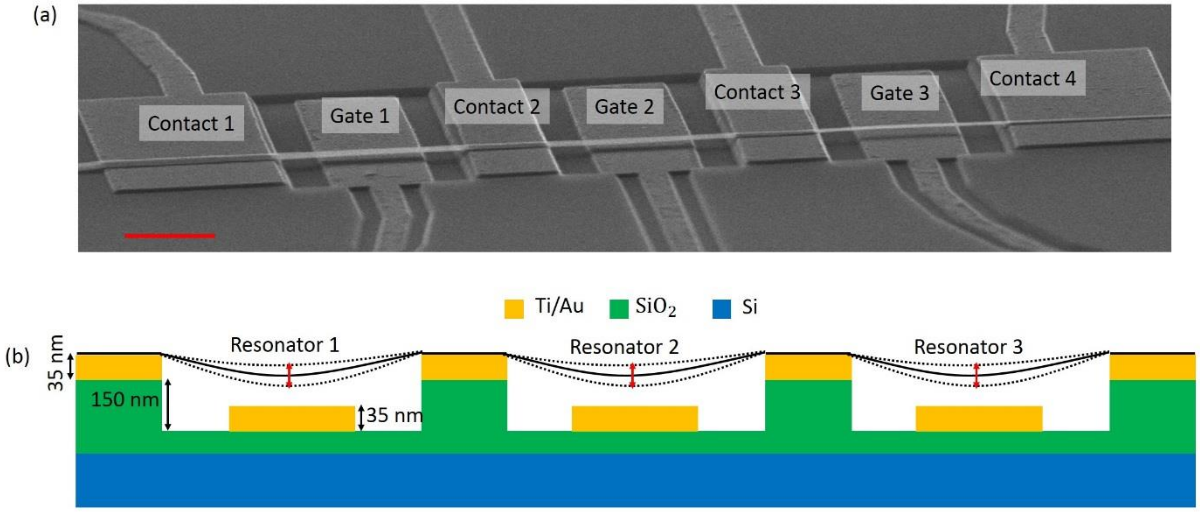

Supplementary Figure 1: Microscopy image and cross-section schematic of the sample. (a) Scanning electron microscope photograph of a typical sample. Four contacts and three bottom gates are fabricated by standard EBL technology. Here contacts 1 and 4 are designed to be 2 μm, while the contacts 2 and 3 are 1 μm in widths. The widths of the bottom gates are designed to be 1.2 μm. The graphene ribbon is about 1μm wide, but it looks narrower because this image was taken from a very large angle (to show the suspended ribbon clearly). (b) Cross-section schematic of the sample. Scale bar is 1 μm.

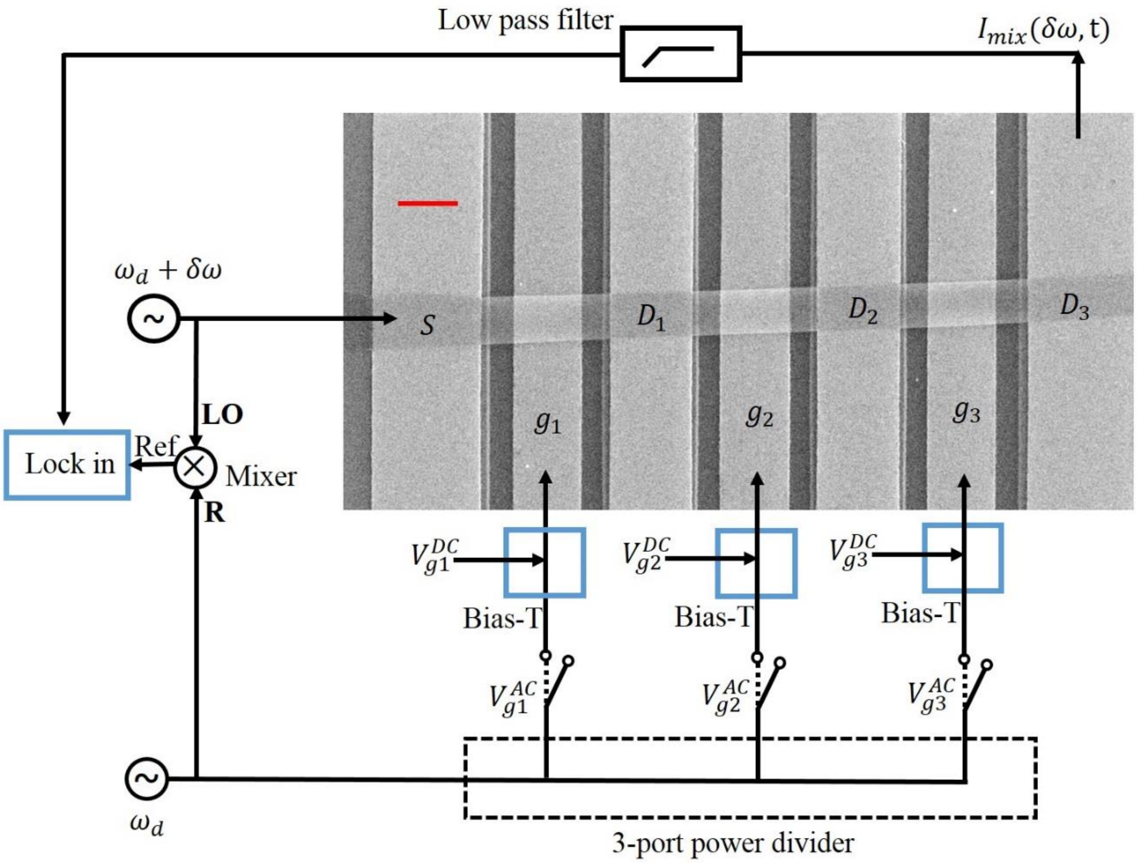

Supplementary Figure 2: Measurement schematic. Two microwave sources (Agilent E8257D) were used in our experiments. Microwave field applied to the source (with frequency $\omega + \delta\omega$) will be mixed with another microwave source applied on the bottom gates (with frequency ω), contributing to new signals at the sum and difference of the original frequencies. We then use a low pass filter with a 1 MHz bandwidth to remove the high frequency component from the signal before sending it to a locking amplifier. A mixer is used to generate the reference signal from the two microwave sources. A three-port power divider is used to divide the microwave field into three components to each bottom gate. Three bias-Tees are used to combine DC and AC signals applied to all bottom gates. Scale bar is 1 μm.

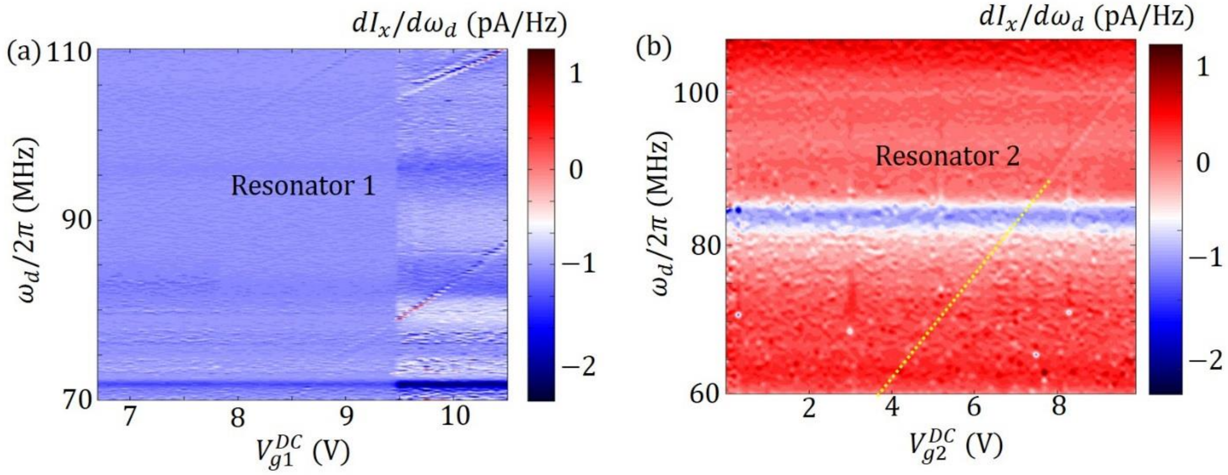

Supplementary Figure 3: Gate tunabilities of resonators R_1 and R_2. (a) Mechanical resonance as a function of gate voltage for R_1. High order resonant modes can be resolved. The resonator can be tuned by gate voltage with a coefficient $df_{01}/d\,V_{g1}^{\mathrm{DC}} \sim 7$ MHz/V. (b) Spectrum as a function of gate voltage for R_2. The gate tuning coefficient is $df_{02}/d\,V_{g2}^{\mathrm{DC}} \sim 6.7$ MHz/V.

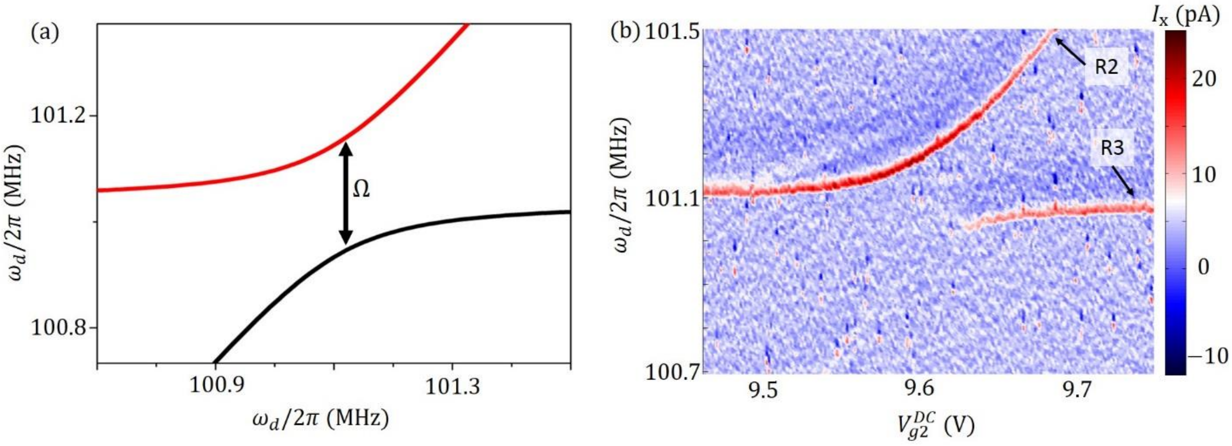

Supplementary Figure 4: Coupling between two mechanical modes. (a) Theory of the two mode coupling. (b) Experimental results of the coupling between R_2 and R_3.

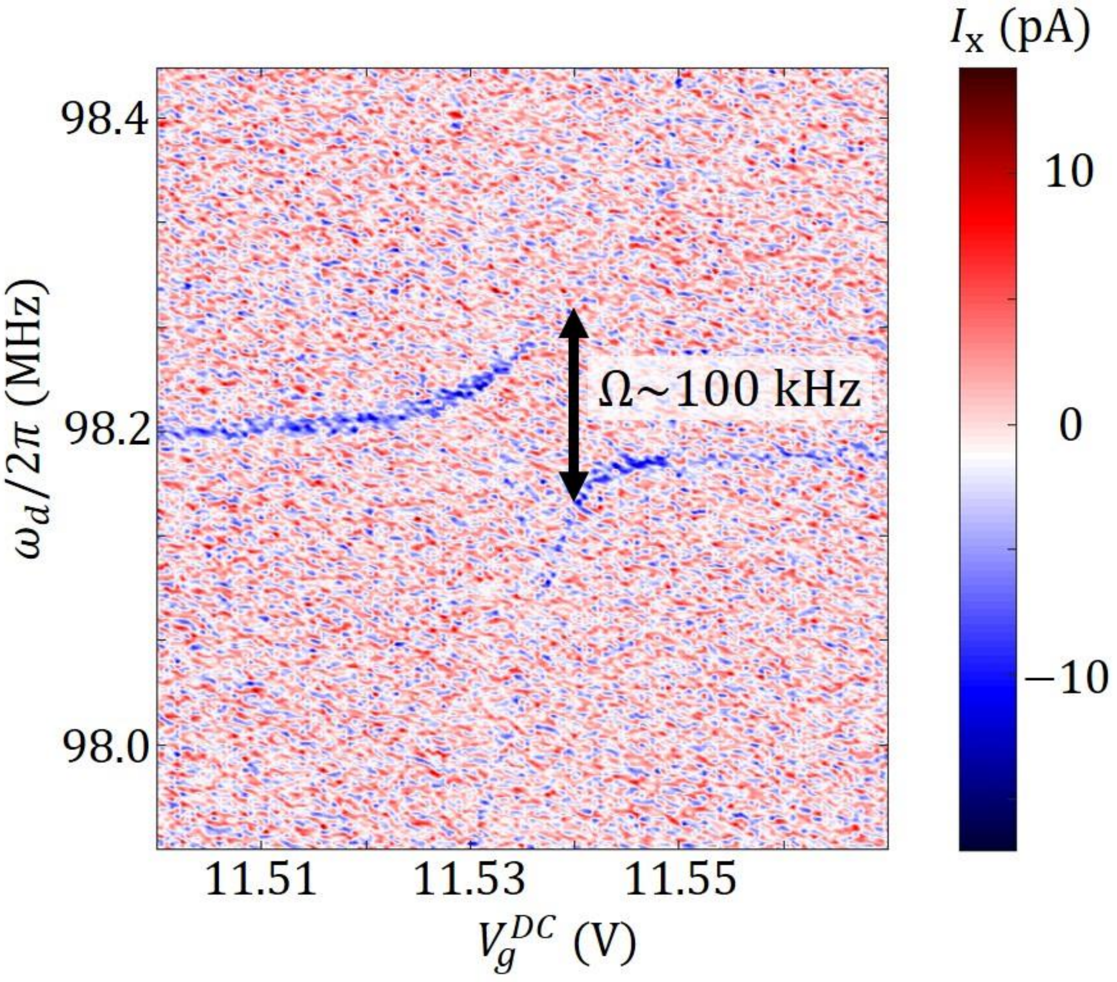

Supplementary Figure 5: Coupling spectrum between neighboring resonators using another device. The parameters of this device have been described in Supplementary Methods and Supplementary Figure 1. In this sample only resonator 1 and resonator 2 can work and the measured coupling strength is about 100 kHz.

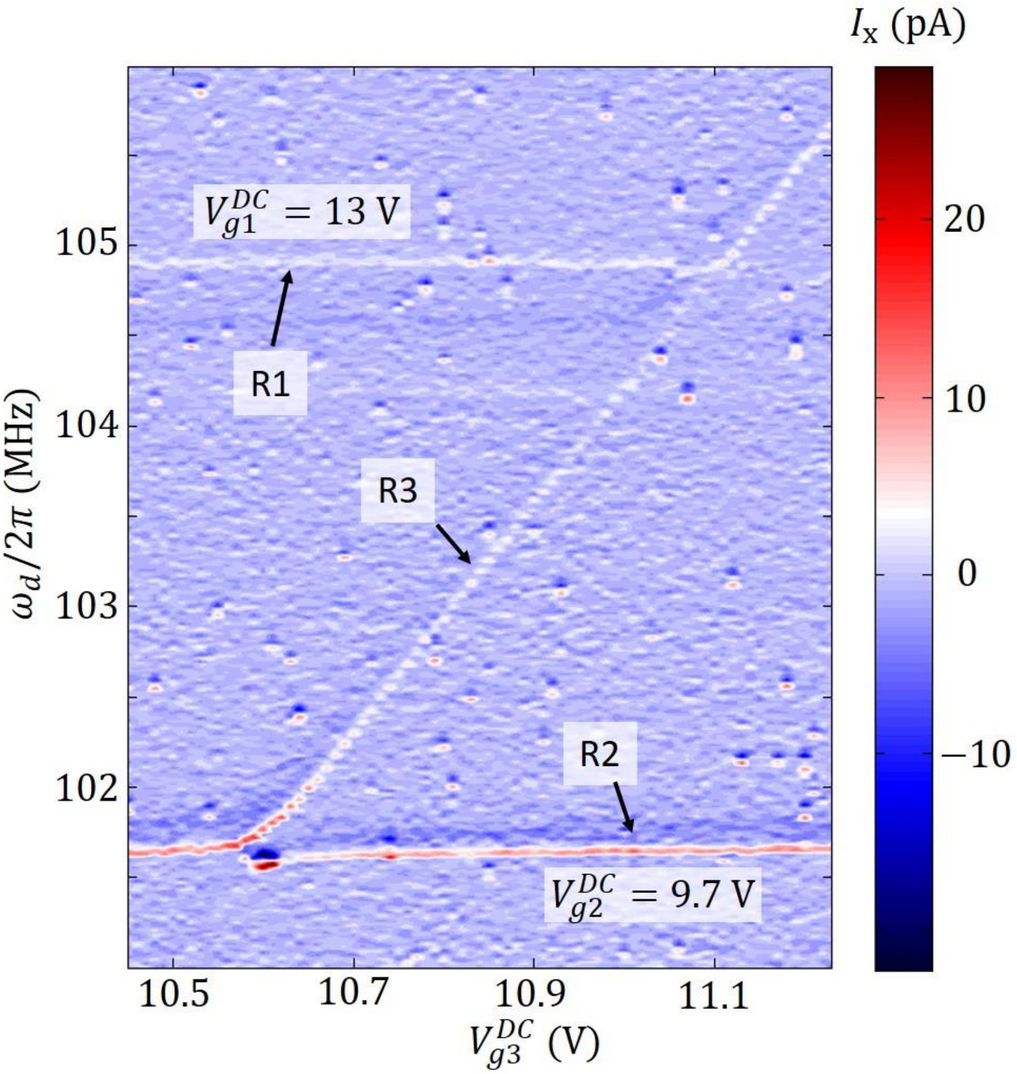

Supplementary Figure 6: Spectrum of the three resonators in a larger range. Here $V_{g1}^{\mathrm{DC}} = 13$ V and $V_{g2}^{\mathrm{DC}} = 9.7$ V. This leads to a detuning of $\Delta_{12} = 3.3$ MHz and very small direct coupling between R_1 and R_3.

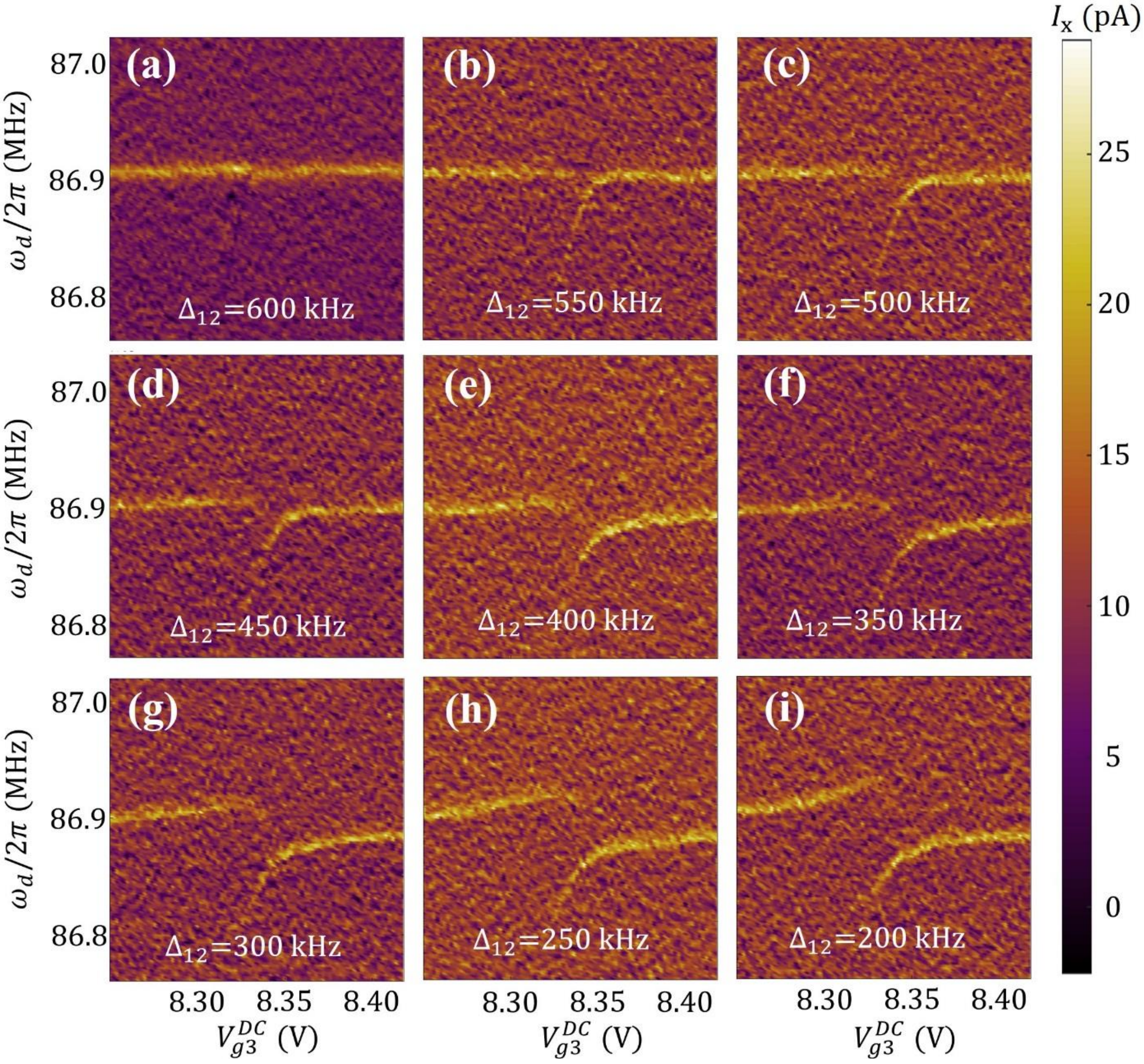

Supplementary Figure 7: Coupling spectrum between R_1 and R_3 at different detuning Δ_{12}. Here we do not include the spectrum of R_2 in these diagrams. And V_{g1}^{DC} is fixed at 10.5 V. From panel (a) to panel (i), V_{g2}^{DC} are 7.62 V, 7.613 V, 7.606 V, 7.599 V, 7.592 V, 7.585 V, 7.578 V, 7.571 V, and 7.564 V, respectively.

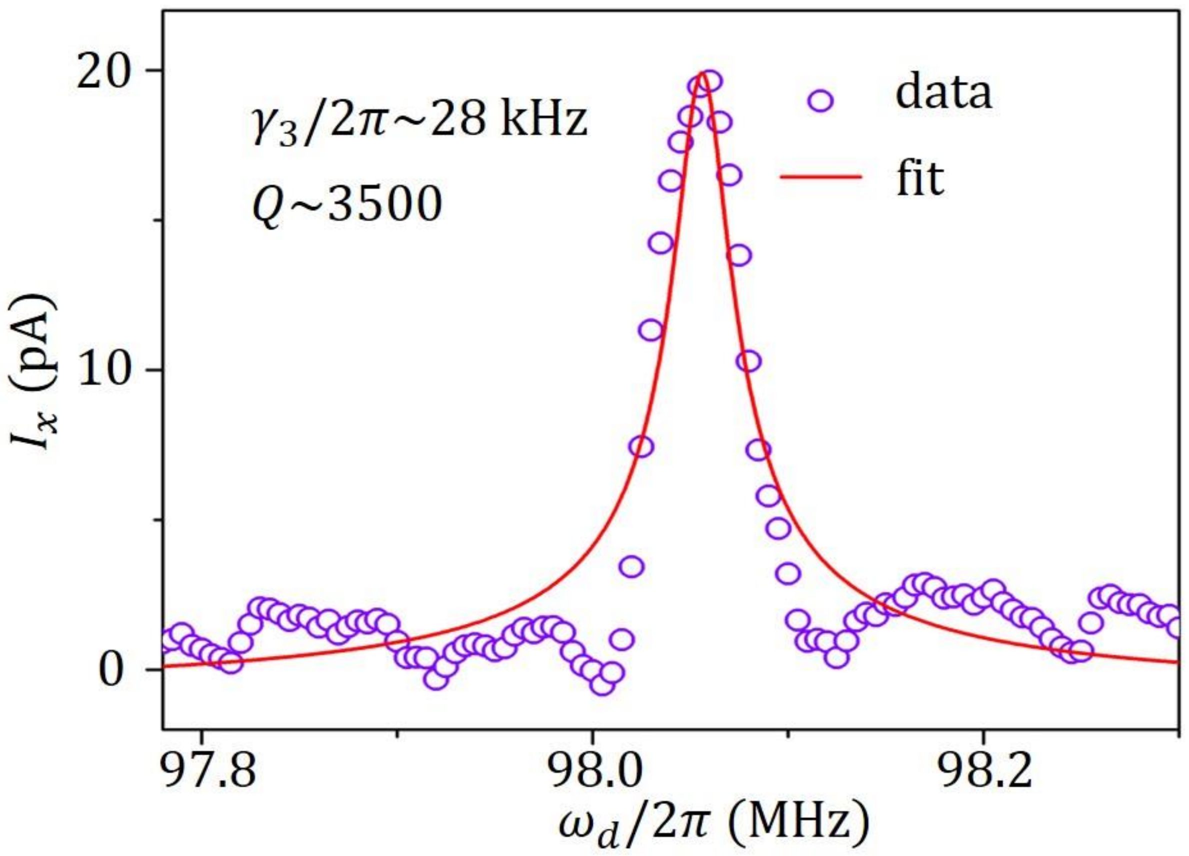

Supplementary Figure 8: Fitting of the mixing current peak. We use equation $I_x = A\frac{\gamma/\omega_0}{\sqrt{(1-\left(\frac{\omega}{\omega_0}\right)^2)^2+(\frac{\omega\gamma}{(\omega_0)^2})^2}}$ to extract the resonant center ω_0 and the linewidth γ of a resonator[3]. Here A is a fitting constant related to the transconductance and driving power and ω is the driving frequency.

Supplementary References:

1 Castellanos-Gomez, A. et al. Deterministic transfer of two-dimensional materials by all-dry viscoelastic stamping. *2D Materials* **1**, 011002 (2014).

2 Chen, C. Y. et al. Graphene mechanical oscillators with tunable frequency. *Nat. Nanotech.* **8**, 923-927 (2013).

3 Sazonova, V. et al. A tunable carbon nanotube electromechanical oscillator. *Nature* **43**, 284-287 (2004).
